# Food Away From Home and Body Mass Index: Insights From a Quantile Regression Analysis of NHANES 2011–2020

**DOI:** 10.1155/bmri/6298673

**Published:** 2026-09-23

**Authors:** Nirajan Budhathoki, Keshab Raj Dahal, Umesh Prasad Bhusal

**Affiliations:** ^1^ Department of Public Health Sciences, Henry Ford Health, Detroit, Michigan, USA, henryford.com; ^2^ Department of Epidemiology and Biostatistics, Michigan State University, East Lansing, Michigan, USA, msu.edu; ^3^ Department of Mathematics, State University of New York Cortland, Cortland, New York, USA, uoh.edu.sa; ^4^ School of Population Health, The University of New South Wales (UNSW), Sydney, Australia, unsw.edu.au

**Keywords:** body mass index, food away from home, NHANES, quantile regression

## Abstract

**Background:**

Food away from home (FAFH) has several health implications and is believed to be a contributing factor to the rise in obesity. However, studies focusing on the impact of FAFH along the distribution of body mass index (BMI) remain scarce. This cross‐sectional study is aimed at using nationally representative survey data over multiple years to examine whether FAFH affects individuals differently based on their BMI.

**Methods:**

Data from the four recent cycles of the US National Health and Nutrition Examination Survey (NHANES) were pooled for analysis. Quantile regression (QR) method was utilized to model the outcome of interest “BMI” and the exposure “FAFH,” adjusted for potential confounders. The use of QR allowed differential effects of FAFH on BMI to be evident that would otherwise be masked using a linear or logistic regression model. Survey weights were applied in all analyses to address the complex sampling procedure of the NHANES.

**Results:**

QR method showed differential effects of FAFH on conditional BMI distribution. For individuals in the lower tail of the BMI distribution (0.05 quantile), three or more FAFH a week was associated with BMI increase of 0.29 kg/m^2^ (95% CI: 0.00, 0.58) compared with less than three FAFH. The effect became stronger at the middle quantiles with the strongest effect observed at the 0.60 quantile, where individuals consuming three or more FAFH a week had a BMI of 0.41 kg/m^2^ (95% CI: 0.12, 0.71) higher than those consuming FAFH less frequently. Although higher consumption of FAFH was associated with increased BMI for individuals in lower to middle quantiles, the effect at the upper tail (0.90 quantile), representing individuals in the highest BMI range, was not statistically significant. Additional investigation at the upper quantiles revealed that the association remained statistically significant at 0.70 quantile, whereas estimates at the 0.80 and 0.95 quantiles remained positive but were not statistically significant.

**Conclusion:**

QR revealed a broadly positive FAFH‐BMI association across the BMI distribution, with statistically significant associations through the 70th percentile and less precise estimates in the upper tail. Although further research is needed to better understand these associations, our findings highlight the importance of considering distributional patterns and statistical uncertainty when evaluating the impact of FAFH consumption on BMI and designing public health interventions.

## 1. Introduction

Food away from home (FAFH) has become increasingly popular due to changing lifestyles, urbanization, and the convenience of ready‐made meals. Studies [1, 2] show that the frequency of eating outside has increased steadily, and many people rely on restaurants, fast food outlets, and takeaways for their daily food. This growing trend has significant public health implications, as meals eaten outside home are often higher in calories, unhealthy fats, added sugars, and sodium while being lower in dietary fiber and essential nutrients like vitamins and minerals [3, 4].

The impact of reduced nutrient quality in FAFH on population health is concerning. Researchers [5, 6] highlight that frequent FAFH consumption is associated with a greater risk of overeating, which can lead to increased body mass index (BMI), a measure of weight relative to height used to assess body weight in population health studies. Moreover, eating outside has been linked to an increased risk of chronic diseases, such as cardiovascular conditions and Type 2 diabetes [7, 8] due to its higher energy density and lower nutritional adequacy [9]. These negative health impacts are particularly concerning in light of the growing trend of FAFH consumption globally, underscoring its contribution to the growing burden of weight‐related health problems.

Certain demographic and socioeconomic groups are disproportionately affected by the impact of FAFH on BMI. Low‐income families and young adults are more likely to depend on FAFH due to financial constraints and the convenience it offers, often resulting in higher consumption of calorie‐dense, nutrient‐poor meals [4, 10]. Additionally, structural barriers such as limited access to affordable, healthy food options and targeted fast‐food marketing place ethnic minorities, particularly Black and Hispanic populations in the United States, at heightened risk of obesity [11–13]. Women, who often face additional economic and time‐related challenges, are also significantly impacted [14].

Currently, there is limited evidence from population studies regarding the impact of FAFH on BMI. Although studies have examined the relationship between FAFH and obesity (a condition characterized by excessive body fat and defined as having a BMI of 30 kg/m^2^ or higher) [6, 15], significant gaps remain that warrant further investigation. A major limitation is the scarcity of large, nationally representative studies that integrate data across multiple years to assess the variability of FAFH consumption across population subgroups and its impact on BMI over time. Using data from multiple years helps account for year‐to‐year differences in socioeconomic factors, food environments, and health‐related policies that could influence both FAFH consumption and BMI. Moreover, it also helps achieve a larger sample size, which increases the power of the analysis [16]. Another common but debatable practice is to model BMI by converting it into distinct categories. Since BMI is a continuous variable, such an approach results in a loss of data granularity [17]. Therefore, statistical methods that preserve the continuous nature of BMI in modeling are warranted. The quantile regression (QR) method [18], which is the focus of this study, offers such an advantage over traditional approaches.

This study uses nationally representative survey data spanning from 2011 to 2020 (until March). Although the data are cross‐sectional, we include multiple survey rounds to help account for temporal variations in the relationship between FAFH and BMI, thereby reducing potential confounding from unobserved period‐specific factors. The aim of this study is to determine whether FAFH affects individuals differently across the BMI distribution by examining data over multiple years, providing deeper insights into its impact on BMI and weight status.

## 2. Methods

### 2.1. Data Source

**Figure 1:**
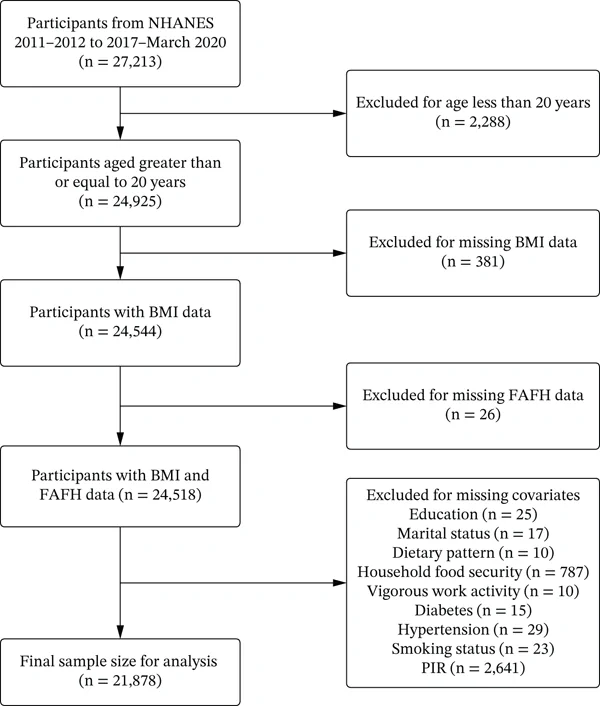
Flowchart of the participant selection for the study. Missing data counts may overlap across variables; therefore, their sum does not equal the total number excluded.

Data were pooled from the four cycles of the National Health and Nutrition Examination Survey (NHANES): 2011–2012, 2013–2014, 2015–2016, and 2017–2020 (until March). NHANES is a nationally representative cross‐sectional study administered by the Centers for Disease Control and Prevention (CDC) in the United States. The survey employs a multistage probability sampling approach to select participants and gathers information on a wide range of topics including demographics, physical examinations, laboratory tests, and dietary assessments. NHANES samples are drawn in four stages: (a) primary sampling units (PSUs) that comprise counties, groups of tracts within counties, or combinations of adjacent counties, (b) segments within PSUs (census blocks or combinations of blocks), (c) dwelling units (households) within segments, and (d) people within households. The PSUs are sampled from all US counties. Data collection is carried out during in‐person interviews followed by physical and health examinations at the Mobile Examination Center. NHANES data are released in a 2‐year cycle; however, the 2019–2020 cycle was not completed due to the COVID‐19 pandemic. Therefore, data collected from 2019 to 2020 (until March) were combined with data from the 2017–2018 cycle to form a nationally representative sample of 2017–2020 prepandemic data. Details about NHANES can be found in the official documentation [19]. Respondents aged 20 years or older were considered in this study. The total number of participants with complete information included in this study was 4778, 5083, 4829, and 7188 for the 2011–2012, 2013–2014, 2015–2016, and 2017–2020 survey cycles, respectively. Figure 1 presents the flow chart of participants′ inclusion in the study.

### 2.2. Study Variables

The outcome variable of interest was BMI; a continuous variable measured in kilograms per square meter. For descriptive purpose, we also created BMI categories based on standard cutoff values for adults aged 20 and older: underweight (< 18.5 kg/m^2^), healthy weight (18.5 kg/m^2^ to < 25 kg/m^2^), overweight (25 kg/m^2^ to < 30 kg/m^2^), and obese (30 kg/m^2^ or greater) [20]. The exposure variable was the frequency of FAFH consumption assessed via the survey question “During the past 7 days, how many meals {did you/did sample person} get that were prepared away from home in places such as restaurants, fast food places, food stands, grocery stores, or from vending machines?” Response to this question was recorded as 0 (none), 1–21 (range of values) or more than 21 meals per week. However, in this study, we categorized the number of FAFH into two categories based on the median value from the data: less than 3, or more than or equal to 3. The decision to use this categorization was based on prior literature studying FAFH [21, 22]. To adjust for potential confounders, several demographic and socioeconomic, behavioral, and health characteristics were included as control variables. Variables selection was based on literature [22–27], limited by their availability in the NHANES databases. Table 1 presents the list of variables and their measurement used in this study.

**Table 1:** List of variables and their measurement.

Variables	Level of measurement
Body mass index	Continuous variable measured in kg/m^2^
FAFH (past week)	Categorical: Less than 3, More than or equal to 3
Survey year	Categorical: 2011–2012, 2013–2014, 2015–2016, 2017–2020 (until March)
Gender	Categorical: Male, Female
Age	Continuous variable measured in years
Race	Categorical: Mexican American, Non‐Hispanic Black, Non‐Hispanic White, Non‐Hispanic Asian, Other Hispanic, Other race—including multiracial
Education	Categorical: Below high school, High school graduate, Some college or more
Marital status	Categorical: Married/living with partner, Never married, Widowed/divorced/separated
Poverty‐to‐income ratio (PIR)	Categorical: Income below poverty line (≤ 1), Income above poverty line (> 1)
Household food security	Categorical: Very low, Low, Marginal, High
Dietary pattern	Categorical: Poor, Fair, Healthy
Smoking	Categorical: Current, Former, Never
Vigorous work activity	Categorical: No, Yes
Diabetes	Categorical: No, Borderline, Yes
High blood pressure	Categorical: No, Yes

Here, we describe a few variables that need an additional description. Education level was releveled into three categories: below high school, high school graduate, some college, or more. The economic status of the participants was assessed using the poverty‐to‐income ratio (PIR). NHANES uses US Department of Health and Human Services poverty guidelines as the poverty measure to calculate this ratio [19]. In the survey, PIR is recorded as a number ranging from 0 to 5. All values greater than 5 are top‐coded 5 because of disclosure concerns. As done by Greenblatt et al. [28], we releveled this ratio into two categories: income below poverty line (PIR less than or equal to 1) and income above poverty line (PIR greater than 1). The overall diet quality was originally recorded in the survey using five categories: poor, fair, good, very good, and excellent. In this study, we reclassified these into three categories: poor, fair, and healthy. Participants′ smoking status was determined through two survey questions. The first question asked if they had smoked at least 100 cigarettes in their lifetime. Respondents who answered “No” were categorized as never smokers. Those who answered “Yes” were further asked if they currently smoke. Participants who responded affirmatively to this second question were classified as current smokers, whereas those who answered “No” were categorized as former smokers. In the survey, participants were considered to perform vigorous work activity if their work involved vigorous activity that causes large increases in breathing or heart rate, such as carrying or lifting heavy loads, digging or construction work for at least 10 min continuously. Responses on diabetes and high blood pressure status were self‐reported by the survey participants.

### 2.3. Statistical Analysis

Descriptive statistics were used to summarize the characteristics of the study sample. Categorical variables were summarized using frequencies and percentages, whereas continuous variables were summarized using median, first quartile (Q1), and third quartile (Q3). The proportion of missing data was low, less than 1% for most variables, except for BMI (1.5%), household food security (3.2%), and PIR (10.6%). Prior to analysis, observations with missing data were excluded assuming missing at random, and a complete case analysis was done on a final analytic sample of 21,878 participants (see Figure 1).

The outcome of interest, BMI, is a continuous variable. However, it is a common practice to categorize BMI and study the odds of being in one of these categories using logistic regression. An alternative approach that preserves the continuous nature of BMI data is to model it using the ordinary least squares (OLS) regression method. However, the OLS method is limited in that it only models the average BMI values as a function of the covariates. On the other hand, the QR method, introduced by Koenker and Bassett [18], overcomes this limitation by modeling the relationship between covariates and quantiles of the outcome of interest.

Let *Y* be the outcome variable (BMI in our study) and *X* be a vector of explanatory variables that includes both the exposure and control variables. The *τ* quantile of *Y* conditional on *X* = *x* can be modeled using the QR model given in Equation (1).

(1) Qyixiτxi=xiTβT,

where $Q_{y_{i}\left|x_{i}\right.}\left(\tau \left|x_{i}\right.\right)$ is the conditional *τ* *t* *h* quantile outcome given *x*_*i*_; *τ* *ϵ* (0, 1) is the *τ* *t* *h* quantile of the BMI. When *τ* = 0.5, we get a median regression model for the BMI. $x_{i}={\left(x_{i1},x_{i2},\cdots .,x_{ip}\right)}^{T}$is the vector of explanatory variables for each individual *i*, and $\beta_{\tau }={\left(\beta_{\tau 0},\beta_{\tau 1},\cdots .,\beta_{\tau p}\right)}^{T}$is the vector of (*p* + 1) regression coefficients at a known *τ* [18, 29].

Studies have shown that the influence of a particular variable may differ across the BMI distribution [25, 30]. Therefore, this study employs QR to examine the effects of FAFH at different points of conditional distribution of the BMI. In particular, we considered the 5th, 30th, 50th, 60th, and 90th percentiles as the quantiles of interest. The percentiles were selected based on the empirical evidence as they correspond to distinct weight classifications within the study sample: The 5th, 30th, 60th, and 90th percentiles represent underweight, healthy weight, overweight, and obese categories with BMI values as outlined in Subsection 2.2. The 50th percentile was included to represent the median BMI value. We further evaluated the pattern of association in the upper quantiles of the BMI distribution by fitting QR models at the 70th, 80th, and 95th percentiles. These additional quantiles were examined to assess whether the lack of statistical significance at the 90th percentile reflected an isolated finding or a broader pattern across the upper tail of the BMI distribution.

To further characterize participants across the BMI distribution, the analytic sample was divided into six segments using the survey‐weighted BMI values corresponding to the 5th, 30th, 60th, and 90th percentiles: ≤ 5th, > 5th–30th, > 30th –50th, > 50th –60th, > 60th –90th, and > 90th percentile (Table S1).

**Figure 2:**
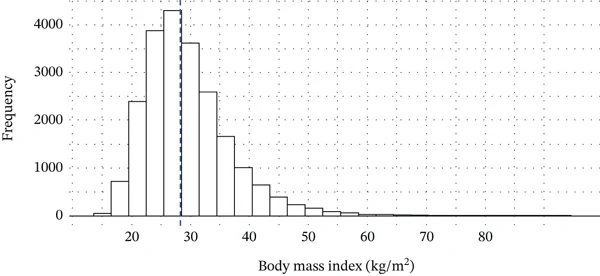
BMI distribution for the study population (NHANES 2011–March 2020). The blue dashed line represents the median BMI value.

Figure 2 presents the distribution of BMI for the study population. It can be observed that the distribution is skewed to the right with few outliers, providing further rationale for using QR that is known to work well in the presence of outliers and nonnormal outcome variable [31]. The change in QR coefficient estimates for FAFH across the distribution of BMI values was plotted. For comparison, we also reported findings from the OLS regression, which models the mean BMI values and serves as a conventional benchmark.

Data were analyzed using R programming language. The *car* package [32] was used to test for the presence of multicollinearity among covariates in the regression models. The test produced generalized variance inflation factor (VIF) values below 2, far below often used threshold of 10 [33], indicating no evidence of multicollinearity (see File S1). Therefore, all covariates were considered for inclusion into the model. The *quantreg* package [34] was used to build QR models. All analyses were weighted using the CDC‐computed sampling weights for individual respondents, which help correct for nonresponse bias often present in survey data. Since data from multiple rounds of the NHANES survey were considered, sampling weights were adjusted according to analytic guidelines from the CDC [35]. The total survey period considered in the study was 9.2 years. Therefore, for all 2‐year survey cycle (2011–2012, 2013–2014, and 2015–2016), the survey weights were adjusted by a factor of 2/9.2. For the 2017–March 2020 cycle, the survey weights were adjusted by a factor of 3.2/9.2. Two‐sided *p* values less than 0.05 were considered statistically significant.

## 3. Results

### 3.1. Descriptive Statistics

Table 2 presents the characteristics of the study population. The median BMI value was 28.2 kg/m^2^. The proportion of participants consuming three or more FAFH during the past week of the survey was slightly higher than those consuming less than 3 FAFH: 51.5% versus 48.5%. The number of females slightly outnumbered the number of males in the study. Almost two‐thirds of the participants were non‐Hispanic White. A total of 64.1% had attended some college or more, and 63.1% were married/living with their partner during the survey time. A majority of the participants (85.1%) had income level above the poverty line. More than 70% of the participants reported both a full household food security and a healthy dietary pattern. Similarly, 18.3% of the participants were current smokers, and 22.9% reported doing some sort of vigorous work activity. Self‐reported diabetes was present in 10.5% of the participants, and almost one‐third reported having high blood pressure.

**Table 2:** Baseline characteristics of the study participants (*n* = 21,878). Frequencies represent unweighted counts; percentages are survey‐weighted estimates based on NHANES sampling design.

Variables	Frequency (%) or median (Q1, Q3)
BMI (kg/m^2^)	28.2 (24.4, 33.0)
BMI category	
Underweight	342 (1.4%)
Healthy weight	5874 (26.9%)
Overweight	6935 (32.2%)
Obese	8727 (39.5%)
FAFH (past week)	
Less than 3	11,921 (48.5%)
More than or equal to 3	9957 (51.5%)
Survey year	
2011–2012	4778 (21.4%)
2013–2014	5083 (21.9%)
2015–2016	4829 (22.0%)
2017–March 2020	7188 (34.7%)
Gender	
Male	10,559 (48.1%)
Female	11,319 (51.9%)
Age (years)	47 (33, 61)
Race	
Mexican American	2703 (8.0%)
Non‐Hispanic Black	5167 (11.1%)
Non‐Hispanic White	8305 (65.9%)
Non‐Hispanic Asian	2658 (5.4%)
Other Hispanic	2234 (6.3%)
Other race—including multiracial	811 (3.3%)
Education	
Below high school	4450 (13.1%)
High school graduate	4920 (22.9%)
Some college or more	12,508 (64.1%)
Marital status	
Married/living with partner	12,873 (63.1%)
Never married	4232 (18.6%)
Widowed/divorced/separated	4773 (18.3%)
PIR	
Income below poverty line	4861 (14.9%)
Income above poverty line	17,017 (85.1%)
Household food security	
Very low	1896 (6.6%)
Low	2954 (9.9%)
Marginal	2803 (10.2%)
High	14,225 (73.3%)
Dietary pattern	
Poor	1,383 (5.6%)
Fair	5,271 (22.3%)
Healthy	15,224 (72.1%)
Smoking	
Current	4212 (18.3%)
Former	5128 (25.0%)
Never	12,538 (56.7%)
Vigorous work activity	
No	17,362 (77.2%)
Yes	4516 (22.9%)
Diabetes	
No	18,276 (87.1%)
Borderline	575 (2.4%)
Yes	3027 (10.5%)
High blood pressure	
No	13,818 (67.4%)
Yes	8060 (32.6%)

Table S1 summarizes participant characteristics across segments of the BMI distribution. Several characteristics varied across the distribution. The prevalence of three or more FAFH consumption increased modestly from 51.0% in the ≤ 5th percentile segment to 55.3% above the 90th percentile. More pronounced differences were observed for dietary and health characteristics: The prevalence of a healthy dietary pattern decreased from 76.6% to 48.4%, whereas diabetes increased from 2.4% to 21.7% and high blood pressure from 15.1% to 49.6% across these respective segments.

### 3.2. Results From OLS and QR

Table 3 summarizes the relationship between FAFH and BMI across different quantiles of the BMI distribution using the OLS as well as QR (see Table S2 for full results). Here, we discuss the adjusted coefficients as they account for the impact of the control variables in the model. The OLS estimate indicates a statistically significant 0.30 kg/m^2^ (95% CI: 0.12, 0.49) higher mean BMI for individuals consuming FAFH three or more times a week compared with those consuming it less frequently, after adjusting for other covariates. This suggests that frequent FAFH consumption is linked to increased BMI on average. A critical assumption of the OLS method, constant error variance (homoscedasticity), was tested visually using the plot of residuals versus fitted values and statistically using studentized Breush–Pagan test. Diagnostic results indicated a violation of the homoscedasticity assumption, further supporting the use of QR (see File S1).

**Table 3:** OLS and QR results for the association between FAFH and BMI (*n* = 21,878).

Estimator	Crude coefficients (95% CI)	Adjusted coefficients (95% CI)
OLS	0.52 (0.33, 0.71) ^∗∗∗^	0.30 (0.12, 0.49) ^∗∗^
0.05 quantile	0.10 (−0.25, 0.45)	0.29 (0.00, 0.58) ^∗^
0.30 quantile	0.40 (0.13, 0.67) ^∗∗^	0.35 (0.12, 0.58) ^∗∗^
0.50 quantile	0.60 (0.30, 0.90) ^∗∗∗^	0.29 (0.03, 0.56) ^∗^
0.60 quantile	0.70 (0.36, 1.00) ^∗∗∗^	0.41 (0.12, 0.71) ^∗∗^
0.70 quantile	0.70 (0.35, 1.05) ^∗∗∗^	0.39 (0.07, 0.71) ^∗^
0.80 quantile	0.80 (0.33, 1.27) ^∗∗∗^	0.33 (−0.04, 0.70)
0.90 quantile	1.00 (0.30, 1.70) ^∗∗^	0.32 (−0.18, 0.81)
0.95 quantile	0.80 (−0.03, 1.63)	0.25 (−0.38, 0.87)

*Note:* Crude coefficients represent association between BMI and FAFH without accounting for covariates. Adjusted coefficients control for the effects of survey year, gender, age, race, education, marital status, PIR, household food security, dietary pattern, smoking, vigorous work activity, diabetes, and high blood pressure, providing independent effect of FAFH on BMI. Regression coefficients are the estimated effects of “Three or more” FAFH during past week of the survey. The reference group consists of those consuming “Less than 3” FAFH.

^∗^ *p* < 0.05,  ^∗∗^ *p* < 0.01,  ^∗∗∗^ *p* < 0.001.

The QR results highlight the differential impact of FAFH consumption across the BMI distribution. The effect is marginal at the lower tail: At 0.05 quantile, three or more FAFH a week was associated with BMI increase of 0.29 kg/m^2^ (95% CI: 0.00, 0.58) compared with less than three FAFH. The effect becomes stronger at the 0.30 quantile where the corresponding estimate of BMI was 0.35 (95% CI: 0.12, 0.58). At the 0.50 quantile, representing the median of the BMI distribution, individuals who reported consuming three or more FAFH a week had a BMI that was 0.29 kg/m^2^ (95% CI: 0.03, 0.56) higher than those consuming FAFH less frequently. This suggests that even among individuals at the median BMI level—typically within the healthy to slightly overweight range—increased frequency of FAFH is significantly associated with greater BMI.

**Figure 3:**
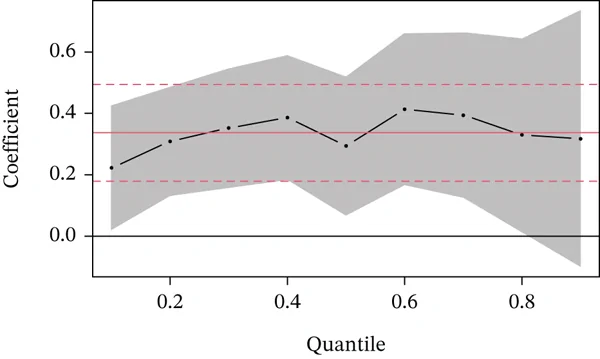
Plot of FAFH effects on BMI from QR, adjusted for covariates and their associated 95% confidence interval (gray shaded region). The solid red line denotes ordinary least squares estimates, and the red dashed lines represent their 95% confidence intervals.

The strongest effect is observed at the 0.60 quantile, which essentially represents the quantile for individuals with overweight. At this quantile, individuals consuming three or more FAFH a week have a BMI of 0.41 kg/m^2^ (95% CI: 0.12, 0.71) higher than those consuming FAFH less frequently. Although a higher consumption of FAFH is associated with increased BMI for individuals in lower to middle quantiles, the effect at the highest (0.90) quantile is not statistically significant. However, it may be noted that the estimate at the 0.90 quantile is similar in magnitude to the OLS estimate and the lower to middle (until 0.50) quantiles. The wider confidence interval at the 0.90 quantile likely reflects greater variability and reduced precision in the tails of the BMI distribution. Figure 3 presents the plot of estimated coefficients for the effect of FAFH across conditional quantiles of BMI, adjusted for the covariates. The figure further illustrates the broadly consistent effect of FAFH along the entire conditional BMI distribution.

Additional analyses at the upper quantiles showed that the association remained statistically significant at the 0.70 quantile (*β* = 0.39, 95% CI: 0.07, 0.71), whereas estimates at the 0.80 (*β* = 0.33, 95% CI: −0.04, 0.70) and 0.95 (*β* = 0.25, 95% CI: −0.38, 0.87) quantiles were not statistically significant. However, the point estimates remained positive and similar in magnitude with confidence intervals widened toward the upper tail of the BMI distribution (see Table S2).

To contextualize the magnitude of the FAFH effect on BMI, results in Table S2 also show that the OLS estimate for having a healthy dietary pattern versus a poor pattern was ‐4.0 (95% CI: −4.3, −3.6), which is approximately 10 times the size of the FAFH effect estimate (0.30, 95% CI: 0.12, 0.49). This contrast highlights that, although FAFH frequency is associated with BMI, other lifestyle factors such as overall diet quality may have an even stronger association with BMI.

## 4. Discussion

This study adds to the growing literature on dietary behaviors and body weight by examining the relationship between FAFH consumption and BMI among US adults. Although several prior studies have evaluated the average effect of FAFH on BMI [36–39], the QR approach used in our study offers insights across the full BMI distribution, capturing nuances that may be masked in mean‐based models. To the best of our knowledge, this study is the first to examine the differential impact of FAFH consumption on BMI using QR in a nationally representative adult population.

The descriptive analysis across BMI distribution segments demonstrates that different portions of the BMI distribution are characterized by distinct demographic, behavioral, and health profiles. Participants in the upper BMI segments had a lower prevalence of healthy dietary patterns and substantially higher prevalences of diabetes and high blood pressure. These findings provide additional context for the QR results and illustrate the value of examining the entire BMI distribution rather than relying solely on mean‐based analysis.

The findings of our study indicate that, after adjusting for demographic, socioeconomic, behavioral, and health characteristics, frequent FAFH consumption is positively associated with BMI across most quantiles with point estimates that were relatively consistent in magnitude. The strongest effect was observed around the 60th percentile, representing individuals with overweight. The association of FAFH with BMI may be explained through several behavioral and psychological mechanisms. One primary factor is portion size, as meals served in restaurants and fast‐food outlets tend to be significantly larger than standard home‐cooked portions, leading to excessive calorie consumption [40, 41]. Additionally, the convenience and accessibility of FAFH play a crucial role in food choices, as individuals with time constraints due to work, family responsibilities, or social commitments often opt for quick, energy‐dense meals that require minimal effort for preparation [42, 43]. Food marketing further reinforces these patterns by promoting highly palatable, calorie‐rich foods through aggressive advertising, pricing strategies, and placement in easily accessible locations [44, 45]. Moreover, psychological factors such as stress and emotional eating can drive individuals toward consuming FAFH, which is often designed to be hyper‐palatable and rewarding, potentially reinforcing overeating behaviors [46, 47]. These mechanisms collectively contribute to higher energy intake, poor diet quality, and ultimately, weight gain, emphasizing the need for public health efforts to address the environmental and behavioral drivers of FAFH consumption.

This study also found that the estimate of the effect of FAFH on BMI was not statistically significant at the upper end of the distribution (0.90 quantile). However, this lack of statistical significance may not be interpreted as an absence of association, as the point estimate was similar to those at the median and the mean. The wider confidence interval at the 0.9 quantile likely reflects reduced precision inherent to QR at the tails due to data sparsity [48]. Examination of the upper BMI distribution provided further context for this finding. The association remained statistically significant at the 0.70 quantile, whereas estimates at the 0.80, 0.90, and 0.95 quantiles were not statistically significant. Importantly, the point estimates remained positive and relatively similar in magnitude across these quantiles, whereas the confidence intervals became progressively wider toward the upper tail. This pattern suggests that the loss of statistical significance at higher quantiles is more consistent with reduced estimation precision than with an absence or reversal of the FAFH‐BMI association.

Previous studies exploring the relationship between FAFH and BMI considered modeling odds for different BMI categories using logistic regression [36–38], or the mean BMI using linear regression [39]. A study conducted in Brazil reported that out‐of‐home eating, defined as the purchase of at least one food or drink item for out‐of‐home consumption during a week period, was associated with overweight and obesity among adults [36]. Overall, out‐of‐home eating was positively associated with overweight (odds ratio = 1.21, 95% CI: 1.10–1.33) and obesity (odds ratio = 1.35, 95% CI: 1.16–1.57) among men, but not women.

In contrast, a study conducted among South Korean adults found that the daily eating‐out rate was positively associated with obesity and overweight in women, but not men [37]. The study reported a graded increase in odds of becoming obese or overweight with higher eating‐out rates among women, particularly who were married. Another study among free‐living US adults indicated that increasing the proportion of either breakfast or dinner eating away from home was associated with increased risk of obesity [38].

Although these studies reported associations between FAFH and BMI categories, none of them examined the differential impact of FAFH on the entire conditional distribution of the BMI. Consequently, the findings we obtained using QR in our study were not evident in the previous studies. In addition to FAFH, we found that overall dietary pattern was strongly associated with BMI in the adjusted models, with individuals reporting a healthy dietary pattern having substantially lower BMI compared with those reporting a poor dietary pattern. The magnitude of this association was markedly larger than the estimated effect of FAFH frequency, underscoring an important role of diet quality in shaping body weight. This finding is consistent with prior evidence linking high‐quality dietary patterns to lower BMI and obesity risk [49]. Together, these results suggest that although frequent FAFH consumption contributes to higher BMI, its effect is modest relative to the effect of overall dietary pattern.

The strength of this study includes the use of nationally representative NHANES data spanning multiple survey cycles, enhancing the generalizability and robustness of the findings. Importantly, the use of QR allowed for a more nuanced assessment of the FAFH‐BMI relationship across the conditional BMI distribution. Those patterns in the relationship would be masked in traditional mean‐based analysis using OLS method as it assumes a uniform effect across all individuals. This methodological advantage has been recognized in previous studies investigating BMI as well as other health outcomes [24, 31, 50, 51]. Furthermore, we adjusted for a range of demographic, socioeconomic, behavioral, and health characteristics in the analysis models, improving the robustness of the findings.

Despite its strengths, our study has some limitations. First, although we considered data from multiple survey cycles to possibly account for the temporal variations, we could not establish causality between FAFH and BMI due to cross‐sectional nature of our study design. Experimental or quasi‐experimental design may help establish causal relationship. Second, the measurement of FAFH relied on self‐reported data, which may introduce recall bias, potentially affecting the accuracy of the estimates. However, the use of a short 7‐day recall period likely minimized recall error, and any misreporting is expected to be limited. Additionally, although we adjusted for several potential confounders, unmeasured factors such as nutritional composition of FAFH meals may have influenced the observed associations. Finally, while dichotomizing FAFH frequency simplified both analysis and interpretation, and aligned with prior literature [21, 22], it may have masked more granular dose‐response relationships [17]. Future research could consider modeling FAFH as a continuous variable or using finer categorizations to advance our understanding of its association with BMI.

## 5. Conclusion

In conclusion, QR showed a broadly positive association between higher FAFH intake and BMI across the conditional BMI distribution. The association remained statistically significant through the 70th percentile, whereas estimates at higher quantiles remained positive but became less precise, with progressively wider confidence intervals. Descriptive analyses also showed that participants in the upper BMI segments had less favorable dietary patterns and substantially higher prevalences of diabetes and high blood pressure, highlighting important differences in population composition across the BMI distribution. Together, these findings demonstrate the value of examining the full BMI distribution and the associated statistical uncertainty when evaluating diet‐related risk factors. We conclude by acknowledging that the QR framework serves as a valuable tool for public health research, enabling the identification of population subgroups that may be differentially affected by risk factors and informing targeted interventions.

## Author Contributions

Nirajan Budhathoki: conceptualization, data curation, formal analysis, methodology, software, writing—original draft, and writing—review and editing. Keshab Raj Dahal: conceptualization, methodology, writing—original draft, and writing—review and editing. Umesh Prasad Bhusal: conceptualization, methodology, supervision, writing—original draft, and writing—review and editing.

## Funding

No funding was received for this manuscript.

## Ethics Statement

Ethical approval was not required for this study as it involved the analysis of publicly available, deidentified secondary data from the National Health and Nutrition Examination Survey (NHANES). NHANES is conducted with approved protocol from the National Center for Health Statistics (NCHS) Ethics Review Board. Informed consent is obtained from all survey participants.

## Conflicts of Interest

The authors declare no conflicts of interest.

## Supporting Information

Additional supporting information can be found online in the Supporting Information section.

## Supporting information


**Supporting Information 1** File S1: Test for multicollinearity and OLS method model diagnostics.

**Supporting Information 2** Table S1: Characteristics of survey participants across segments of the BMI distribution.

**Supporting Information 3** Table S2: Full results from the OLS and quantile regression.

## Data Availability

Data from the National Health and Nutrition Examination Survey (NHANES) are publicly available online at https://wwwn.cdc.gov/nchs/nhanes/default.aspx.
