# Supplementary material for: Food Away From Home and Body Mass Index: Insights From a Quantile Regression Analysis of NHANES 2011–2020
Source: Biomed Res Int. 2026 Sep 23;2026:6298673. doi: 10.1155/bmri/6298673 (PMC13599368; doi:10.1155/bmri/6298673)
Supplement: Supplementary file 1 [file BMRI-2026-6298673-s001.docx]

**Supplementary Table S1**

**Characteristics of survey participants across segments of the BMI distribution (n = 21,878)**

| **Characteristic** | **≤5th percentile**  **n = 1,215¹** | **>5th–30th percentile**  **n = 5,417** | **>30th–50th percentile**  **n = 4,300** | **>50th–60th percentile**  **n = 2,219** | **>60th–90th percentile**  **n = 6,502** | **>90th percentile**  **n = 2,225** |
| --- | --- | --- | --- | --- | --- | --- |
| **FAFH** |  |  |  |  |  |  |
| Less than 3 | 660 (49.0%) | 3,062 (51.0%) | 2,427 (50.2%) | 1,238 (48.0%) | 3,430 (46.6%) | 1,104 (44.7%) |
| Three or more | 555 (51.0%) | 2,355 (49.0%) | 1,873 (49.8%) | 981 (52.0%) | 3,072 (53.4%) | 1,121 (55.3%) |
| **Gender** |  |  |  |  |  |  |
| Female | 765 (67.6%) | 2,735 (54.4%) | 1,882 (44.7%) | 994 (45.3%) | 3,453 (49.6%) | 1,490 (66.0%) |
| Male | 450 (32.4%) | 2,682 (45.6%) | 2,418 (55.3%) | 1,225 (54.7%) | 3,049 (50.4%) | 735 (34.0%) |
| Age | 37 (25.0, 58.0) | 43 (29.0, 59.0) | 49 (35.0, 63.0) | 49 (36.0, 62.0) | 49 (36.0, 62.0) | 46 (33.0, 58.0) |
| **Race** |  |  |  |  |  |  |
| Mexican American | 62 (4.2%) | 434 (5.3%) | 537 (7.9%) | 348 (9.6%) | 1,032 (10.4%) | 290 (8.5%) |
| Non-Hispanic Asian | 303 (11.7%) | 1,250 (10.0%) | 566 (5.6%) | 212 (4.3%) | 304 (2.2%) | 23 (0.6%) |
| Non-Hispanic Black | 273 (11.6%) | 1,033 (9.0%) | 870 (9.1%) | 467 (9.9%) | 1,734 (12.3%) | 790 (17.4%) |
| Non-Hispanic White | 443 (62.8%) | 2,079 (67.6%) | 1,697 (67.9%) | 853 (66.0%) | 2,399 (64.3%) | 834 (63.6%) |
| Other Hispanic | 91 (6.4%) | 428 (4.9%) | 497 (6.9%) | 267 (7.3%) | 764 (7.0%) | 187 (5.7%) |
| Other Race - Including Multi-Racial | 43 (3.3%) | 193 (3.2%) | 133 (2.5%) | 72 (3.0%) | 269 (3.8%) | 101 (4.2%) |
| **Education** |  |  |  |  |  |  |
| Below high school | 216 (11.5%) | 1,012 (12.4%) | 923 (13.3%) | 495 (14.2%) | 1,402 (13.7%) | 402 (12.1%) |
| High school graduate | 259 (22.4%) | 1,101 (19.5%) | 929 (21.6%) | 500 (22.8%) | 1,560 (25.5%) | 571 (26.4%) |
| Some college or more | 740 (66.1%) | 3,304 (68.2%) | 2,448 (65.1%) | 1,224 (63.0%) | 3,540 (60.8%) | 1,252 (61.4%) |
| **Marital Status** |  |  |  |  |  |  |
| Married/Living with Partner | 558 (48.9%) | 3,125 (59.7%) | 2,743 (68.4%) | 1,393 (68.2%) | 3,848 (64.1%) | 1,206 (59.9%) |
| Never Married | 396 (33.8%) | 1,256 (23.9%) | 658 (14.3%) | 314 (12.9%) | 1,093 (15.6%) | 515 (21.0%) |
| Widowed/Divorced/Separated | 261 (17.3%) | 1,036 (16.5%) | 899 (17.2%) | 512 (18.9%) | 1,561 (20.3%) | 504 (19.1%) |
| **PIR** |  |  |  |  |  |  |
| Income above poverty line | 865 (78.0%) | 4,307 (85.7%) | 3,435 (86.7%) | 1,718 (85.4%) | 5,030 (85.3%) | 1,662 (82.4%) |
| Income below poverty line | 350 (22.0%) | 1,110 (14.3%) | 865 (13.3%) | 501 (14.6%) | 1,472 (14.7%) | 563 (17.6%) |
| **Household food security** |  |  |  |  |  |  |
| Full | 803 (71.0%) | 3,799 (77.1%) | 2,949 (77.0%) | 1,445 (74.7%) | 4,029 (70.6%) | 1,200 (63.9%) |
| Low | 154 (10.4%) | 579 (7.7%) | 537 (8.7%) | 309 (9.9%) | 973 (11.1%) | 402 (13.9%) |
| Marginal | 154 (10.9%) | 608 (8.8%) | 528 (9.4%) | 287 (9.7%) | 899 (11.3%) | 327 (11.8%) |
| Very low | 104 (7.7%) | 431 (6.4%) | 286 (4.9%) | 178 (5.6%) | 601 (7.0%) | 296 (10.5%) |
| **Dietary pattern** |  |  |  |  |  |  |
| Poor healthy | 73 (5.3%) | 196 (3.1%) | 185 (3.5%) | 116 (5.1%) | 508 (7.1%) | 305 (12.8%) |
| Fair healthy | 217 (18.1%) | 874 (14.8%) | 875 (17.8%) | 537 (22.7%) | 1,865 (26.7%) | 903 (38.7%) |
| Healthy | 925 (76.6%) | 4,347 (82.1%) | 3,240 (78.7%) | 1,566 (72.3%) | 4,129 (66.2%) | 1,017 (48.4%) |
| **Smoking** |  |  |  |  |  |  |
| Current | 378 (28.9%) | 1,131 (20.4%) | 822 (18.2%) | 385 (16.0%) | 1,119 (16.3%) | 377 (16.2%) |
| Former | 155 (14.4%) | 1,067 (21.2%) | 1,051 (25.4%) | 583 (26.5%) | 1,704 (28.5%) | 568 (27.9%) |
| Never | 682 (56.7%) | 3,219 (58.5%) | 2,427 (56.5%) | 1,251 (57.5%) | 3,679 (55.2%) | 1,280 (56.0%) |
| **Vigorous work activity** | 193 (18.5%) | 1,044 (21.7%) | 860 (23.3%) | 493 (23.8%) | 1,447 (24.4%) | 479 (21.0%) |
| **Diabetes** |  |  |  |  |  |  |
| No | 1,155 (95.7%) | 4,916 (94.0%) | 3,688 (89.7%) | 1,847 (87.7%) | 5,094 (82.2%) | 1,576 (73.9%) |
| Borderline | 15 (1.9%) | 105 (1.5%) | 98 (1.9%) | 61 (2.8%) | 202 (2.6%) | 94 (4.5%) |
| Yes | 45 (2.4%) | 396 (4.4%) | 514 (8.4%) | 311 (9.5%) | 1,206 (15.2%) | 555 (21.7%) |
| **Hypertension** |  |  |  |  |  |  |
| No | 989 (84.9%) | 4,114 (80.6%) | 2,793 (69.3%) | 1,367 (66.7%) | 3,530 (57.9%) | 1,025 (50.4%) |
| Yes | 226 (15.1%) | 1,303 (19.4%) | 1,507 (30.7%) | 852 (33.3%) | 2,972 (42.1%) | 1,200 (49.6%) |

¹Values are n (unweighted) with survey-weighted percentages, or survey-weighted median (Q1, Q3), as appropriate.
