# Supplementary material for: Food Away From Home and Body Mass Index: Insights From a Quantile Regression Analysis of NHANES 2011–2020
Source: Biomed Res Int. 2026 Sep 23;2026:6298673. doi: 10.1155/bmri/6298673 (PMC13599368; doi:10.1155/bmri/6298673)
Supplement: Supplementary file 2 [file BMRI-2026-6298673-s002.docx]

**Supplementary File S1**

**Test for multicollinearity**

| Predictor variable | Generalized Variance Inflation Factor (GVIF) | Degree of freedom |
| --- | --- | --- |
| Food away from home (FAFH) | 1.12 | 1 |
| Gender | 1.11 | 1 |
| Age | 1.74 | 1 |
| Race | 1.32 | 5 |
| Education | 1.29 | 2 |
| Marital status | 1.38 | 2 |
| Poverty-to-income ratio (PIR) | 1.26 | 1 |
| Household food security | 1.34 | 3 |
| Dietary pattern | 1.14 | 2 |
| Smoking | 1.22 | 2 |
| Vigorous work activity | 1.10 | 1 |
| Diabetes | 1.14 | 2 |
| High blood pressure | 1.28 | 1 |
| Survey year | 1.03 | 3 |

**OLS method model diagnostics**

Figure: Plot of residuals vs fitted values for the OLS.

The figure shows increasing variance of the residuals with higher fitted BMI values, indicating heteroscedasticity. This visual pattern is further supported by studentized Breush-Pagan test (BP = 210,161,906, df = 27, p-value < 0.0001), which provided strong statistical evidence against the assumption of homoscedasticity.
