# Supplementary material for: Food Away From Home and Body Mass Index: Insights From a Quantile Regression Analysis of NHANES 2011–2020
Source: Biomed Res Int. 2026 Sep 23;2026:6298673. doi: 10.1155/bmri/6298673 (PMC13599368; doi:10.1155/bmri/6298673)
Supplement: Supplementary file 3 [file BMRI-2026-6298673-s003.docx]

**Supplementary Table S2**

**Full results from the OLS and quantile regression models (n = 21,878)**

| **Predictor** | **OLS** | **0.05 quantile** | **0.30 quantile** | **0.50 quantile** | **0.60 quantile** | **0.70 quantile** | **0.80 quantile** | **0.90 quantile** | **0.95 quantile** |  |
| --- | --- | --- | --- | --- | --- | --- | --- | --- | --- | --- |
| **Survey year** | | | | | | | | | | |
| 2011-2012 | — | — | — | — | — | — | — | — | — |  |
| 2013-2014 | 0.26 (0.00, 0.52) | -0.06 (-0.44, 0.31) | -0.09 (-0.39, 0.20) | 0.19 (-0.16, 0.54) | 0.23 (-0.14, 0.59) | 0.37 (0.00, 0.74) | 0.24 (-0.24, 0.73) | 0.76 (0.11, 1.4) | 1.06 (0.22, 1.90) |  |
| 2015-2016 | 0.41 (0.14, 0.67) | 0.05 (-0.37, 0.48) | 0.14 (-0.17, 0.45) | 0.31 (-0.04, 0.67) | 0.37 (-0.02, 0.76) | 0.51 (0.07, 0.95) | 0.67 (0.12, 1.22) | 0.9 (0.21, 1.6) | 1.00 (0.21, 1.78) |  |
| 2017-March 2020 | 0.71 (0.47, 0.95) | 0.03 (-0.39, 0.45) | 0.28 (-0.01, 0.58) | 0.63 (0.27, 1.0) | 0.71 (0.32, 1.1) | 1.05 (0.61, 1.49) | 1.08 (0.59, 1.56) | 1.3 (0.65, 2.0) | 1.05 (0.25, 1.84) |  |
| **Gender** | | | | | | | | | | |
| Female | — | — | — | — | — | — | — | — | — |  |
| Male | -0.66 (-0.85, -0.48) | 1.2 (0.87, 1.5) | 0.57 (0.34, 0.80) | -0.11 (-0.38, 0.17) | -0.44 (-0.74, -0.14) | -0.97 (-1.30, -0.65) | -1.70 (-2.09, -1.30) | -2.8 (-3.3, -2.3) | -3.28 (-3.97, -2.58) |  |
| Age | -0.03 (-0.04, -0.02) | 0.01 (0.00, 0.02) | -0.01 (-0.01, 0.00) | -0.02 (-0.03, -0.01) | -0.03 (-0.04, -0.02) | -0.04 (-0.05, -0.03) | -0.05 (-0.07, -0.04) | -0.07 (-0.09, -0.05) | -0.09 (-0.11, -0.07) |  |
| **Race** | | | | | | | | | | |
| Mexican American | — | — | — | — | — | — | — | — | — |  |
| Non-Hispanic Asian | -4.5 (-5.0, -4.0) | -2.4 (-2.8, -2.1) | -3.8 (-4.1, -3.4) | -4.3 (-4.7, -4.0) | -4.4 (-4.8, -4.1) | -4.59 (-5.04, -4.14) | -5.06 (-5.51, -4.61) | -6.0 (-6.7, -5.3) | -6.71 (-7.91, -5.50) |  |
| Non-Hispanic Black | 0.32 (-0.09, 0.74) | -1.1 (-1.5, -0.75) | -0.63 (-1.0, -0.26) | -0.01 (-0.41, 0.39) | 0.16 (-0.23, 0.55) | 0.82 (0.32, 1.32) | 1.21 (0.71, 1.72) | 1.5 (0.80, 2.2) | 2.02 (0.78, 3.26) |  |
| Non-Hispanic White | -0.81 (-1.2, -0.47) | -1.2 (-1.6, -0.83) | -1.5 (-1.8, -1.1) | -1.2 (-1.6, -0.81) | -0.91 (-1.3, -0.54) | -0.59 (-1.05, -0.13) | -0.44 (-0.91, 0.03) | -0.16 (-0.84, 0.52) | 0.07 (-1.17, 1.31) |  |
| Other Hispanic | -0.76 (-1.2, -0.30) | -0.55 (-1.0, -0.06) | -0.72 (-1.2, -0.26) | -0.79 (-1.2, -0.42) | -0.83 (-1.3, -0.38) | -0.46 (-0.96, 0.04) | -0.53 (-1.11, 0.05) | -0.78 (-1.6, 0.10) | -0.74 (-1.93, 0.45) |  |
| Other Race - Including Multi-Racial | -0.06 (-0.64, 0.52) | -1 (-1.6, -0.45) | -0.9 (-1.7, -0.05) | -0.39 (-1.4, 0.58) | 0.19 (-0.89, 1.3) | 0.63 (0.06, 1.20) | 0.77 (-0.44, 1.98) | 0.27 (-1.1, 1.6) | 0.85 (-1.92, 3.62) |  |
| **Education** | | | | | | | | | | |
| Below high school | — | — | — | — | — | — | — | — | — |  |
| High school graduate | 0.87 (0.56, 1.2) | 0.14 (-0.35, 0.62) | 0.71 (0.34, 1.1) | 0.84 (0.46, 1.2) | 0.87 (0.45, 1.3) | 1.27 (0.81, 1.73) | 1.39 (0.87, 1.91) | 1.3 (0.67, 2.0) | 0.92 (-0.13, 1.96) |  |
| Some college or more | 0.58 (0.29, 0.87) | 0.34 (-0.12, 0.79) | 0.46 (0.16, 0.77) | 0.36 (0.04, 0.68) | 0.31 (-0.03, 0.65) | 0.54 (0.12, 0.95) | 0.82 (0.36, 1.28) | 1.1 (0.45, 1.8) | 0.87 (-0.11, 1.86) |  |
| **Marital Status** | | | | | | | | | | |
| Married/Living with Partner | — | — | — | — | — | — | — | — | — |  |
| Never Married | -1 (-1.3, -0.76) | -1.3 (-1.6, -0.90) | -1.7 (-2.0, -1.3) | -1.4 (-1.9, -1.0) | -1 (-1.4, -0.59) | -0.88 (-1.39, -0.37) | -0.64 (-1.20, -0.09) | 0.06 (-0.58, 0.70) | 0.25 (-1.04, 1.53) |  |
| Widowed/Divorced/Separated | -0.44 (-0.68, -0.20) | -0.46 (-0.81, -0.11) | -0.28 (-0.60, 0.04) | -0.12 (-0.47, 0.23) | -0.02 (-0.34, 0.30) | -0.36 (-0.75, 0.03) | -0.52 (-0.98, -0.05) | -0.64 (-1.3, -0.01) | -0.83 (-1.76, 0.10) |  |
| **PIR** | | | | | | | | | | |
| Income above poverty line | — | — | — | — | — | — | — | — | — |  |
| Income below poverty line | -0.27 (-0.55, 0.00) | -0.46 (-0.79, -0.12) | -0.28 (-0.61, 0.06) | -0.18 (-0.51, 0.15) | -0.13 (-0.51, 0.25) | -0.18 (-0.57, 0.22) | -0.19 (-0.64, 0.26) | -0.38 (-0.95, 0.18) | -0.03 (-1.09, 1.02) |  |
| **Household food security** | | | | | | | | | | |
| Full | — | — | — | — | — | — | — | — | — |  |
| Low | 0.91 (0.59, 1.2) | 0.06 (-0.32, 0.45) | 0.61 (0.20, 1.0) | 0.83 (0.43, 1.2) | 1 (0.60, 1.5) | 1.13 (0.62, 1.64) | 1.27 (0.73, 1.81) | 1.5 (0.78, 2.3) | 1.90 (1.14, 2.65) |  |
| Marginal | 0.72 (0.42, 1.0) | -0.16 (-0.79, 0.46) | 0.44 (0.03, 0.86) | 0.72 (0.28, 1.2) | 0.77 (0.31, 1.2) | 0.81 (0.37, 1.24) | 0.91 (0.36, 1.46) | 1.2 (0.31, 2.1) | 1.81 (0.75, 2.88) |  |
| Very low | 1 (0.63, 1.4) | -0.04 (-0.50, 0.41) | 0.18 (-0.30, 0.66) | 0.19 (-0.35, 0.73) | 0.56 (-0.19, 1.3) | 1.37 (0.56, 2.18) | 2.02 (1.12, 2.91) | 3 (2.0, 4.0) | 2.94 (0.90, 4.98) |  |
| **Dietary pattern** | | | | | | | | | | |
| Poor healthy | — | — | — | — | — | — | — | — | — |  |
| Fair healthy | -1.3 (-1.8, -0.94) | 0.42 (-0.72, 1.6) | -0.89 (-1.6, -0.17) | -1.2 (-1.9, -0.51) | -1.6 (-2.5, -0.82) | -2.08 (-2.81, -1.36) | -2.06 (-3.07, -1.05) | -2.6 (-4.0, -1.2) | -3.62 (-5.29, -1.95) |  |
| Healthy | -4 (-4.3, -3.6) | -0.4 (-1.5, 0.73) | -2.6 (-3.3, -1.9) | -3.7 (-4.4, -3.1) | -4.4 (-5.2, -3.6) | -5.11 (-5.75, -4.47) | -5.57 (-6.54, -4.61) | -6.4 (-7.7, -5.1) | -7.62 (-9.26, -5.99) |  |
| **Smoking** | | | | | | | | | | |
| Current | — | — | — | — | — | — | — | — | — |  |
| Former | 2.2 (1.9, 2.5) | 2 (1.5, 2.4) | 1.9 (1.5, 2.3) | 2 (1.6, 2.4) | 2.3 (1.9, 2.7) | 2.36 (1.8, 2.93) | 2.33 (1.80, 2.86) | 2.3 (1.6, 3.0) | 2.3 (1.44, 3.15) |  |
| Never | 1.7 (1.4, 1.9) | 1.5 (1.1, 1.9) | 1.5 (1.1, 1.8) | 1.6 (1.2, 1.9) | 1.7 (1.3, 2.0) | 1.59 (1.09, 2.08) | 1.43 (0.95, 1.91) | 1.5 (0.81, 2.2) | 1.88 (1.23, 2.53) |  |
| **Vigorous work activity** | | | | | | | | | | |
| No | — | — | — | — | — | — | — | — | — |  |
| Yes | -0.07 (-0.29, 0.15) | 0.31 (0.01, 0.62) | 0.3 (0.02, 0.58) | 0.26 (-0.06, 0.58) | 0.1 (-0.24, 0.44) | -0.17 (-0.58, 0.24) | -0.46 (-0.87, -0.04) | -0.28 (-0.97, 0.41) | -0.37 (-1.07, 0.32) |  |
| **FAFH** | | | | | | | | | | |
| Less than 3 | — | — | — | — | — | — | — | — | — |  |
| Three or more | 0.3 (0.12, 0.49) | 0.29 (0.00, 0.58) | 0.35 (0.12, 0.58) | 0.29 (0.03, 0.56) | 0.41 (0.12, 0.71) | 0.39 (0.07, 0.71) | 0.33 (-0.04, 0.70) | 0.32 (-0.18, 0.81) | 0.25 (-0.38, 0.87) |  |
| **Diabetes** | | | | | | | | | | |
| No | — | — | — | — | — | — | — | — | — |  |
| Borderline | 2.3 (1.7, 2.9) | 0.36 (-1.8, 2.5) | 1.3 (0.40, 2.2) | 1.6 (0.56, 2.6) | 2.4 (0.71, 4.1) | 2.60 (1.56, 3.64) | 2.82 (2.28, 3.36) | 3.1 (0.19, 6.0) | 4.57 (-0.19, 9.33) |  |
| Yes | 3.1 (2.8, 3.4) | 1.5 (0.99, 2.1) | 2.4 (2.0, 2.9) | 2.8 (2.4, 3.3) | 3 (2.6, 3.4) | 3.26 (2.79, 3.74) | 3.61 (2.99, 4.23) | 4.3 (3.6, 5.1) | 4.61 (3.06, 6.16) |  |
| **High blood pressure** | | | | | | | | | | |
| No | — | — | — | — | — | — | — | — | — |  |
| Yes | 2.8 (2.6, 3.0) | 1.5 (1.1, 1.8) | 2.3 (2.1, 2.6) | 2.7 (2.3, 3.0) | 2.9 (2.6, 3.2) | 3.11 (2.75, 3.47) | 3.21 (2.80, 3.62) | 3.4 (2.9, 4.0) | 3.93 (3.17, 4.69) |  |

Values are coefficient (95% CI). Reference categories are indicated by an em dash (—).
